# Can ChatGPT Recognize Its Own Writing in Scientific Abstracts?

**DOI:** 10.7759/cureus.88774

**Published:** 2025-07-25

**Authors:** Paul Sebo

**Affiliations:** 1 Internal Medicine, University Institute for Primary Care, Geneva University Hospital, Geneva, CHE

**Keywords:** ai, artificial intelligence, authorship, chatgpt, research integrity, scientific writing

## Abstract

Background: With the growing use of generative AI in scientific writing, distinguishing between AI-generated and human-authored content has become a pressing challenge. It remains unclear whether ChatGPT (OpenAI, San Francisco, CA) can accurately and consistently recognize its own output.

Methods: We randomly selected 100 research articles published in 2000, before the advent of generative AI, from 10 high-impact internal medicine journals. For each article, a structured abstract was generated using ChatGPT-4.0 based on the full PDF. The original and AI-generated abstracts (n = 200) were then evaluated twice by ChatGPT-4.0, which was asked to rate the likelihood of authorship on a 0-10 scale (0 = definitely human, 10 = definitely ChatGPT, 5 = undetermined). Classifications of 0-4 were considered human, and 6-10 were considered AI generated.

Results: Misclassification rates were high in both rounds (49% and 47.5%). No abstract received a score of 5. Score distributions overlapped substantially between groups, with no statistically significant difference (Wilcoxon p-value = 0.93 and 0.21). Cohen’s kappa for binary classification was 0.33 (95% CI: 0.19-0.46) and weighted kappa on the 0-10 scale was 0.24 (95% CI: 0.15-0.34), both reflecting poor agreement.

Conclusion: ChatGPT-4.0 cannot reliably identify whether a scientific abstract was written by itself or by humans. More robust external tools are needed to ensure transparency in academic authorship.

## Introduction

Large language models (LLMs), such as ChatGPT (OpenAI, San Francisco, CA), are increasingly used across domains to generate fluent, human-like text. In medicine, ChatGPT has been applied to tasks such as answering patient questions, supporting clinical decision-making, summarizing research, and assisting in manuscript writing [1-6]. While these capabilities are promising, they raise concerns in scientific publishing, particularly around transparency, authorship attribution, and the potential misuse of AI-generated content [7-9].

The scientific community is now facing the challenge of distinguishing between human-written and AI-generated content. Several AI-detection tools have emerged (e.g., GPTZero and Originality.ai), but their accuracy is inconsistent and often unvalidated [10,11]. Human reviewers also struggle to identify AI-generated content with reliable accuracy [4,12-15].

Given this context, an open question is whether ChatGPT can recognize its own output. If it could, it might serve as a self-checking tool for researchers, editors, reviewers, and institutions to flag potentially AI-generated content. This is particularly relevant as publishers begin to develop guidelines and safeguards for AI use in manuscript preparation. To our knowledge, no peer-reviewed studies have directly assessed ChatGPT’s ability to identify its own writing. A recent preprint introduced the concept of self-detection, evaluating whether language models could determine if a given text was generated by themselves. The authors tested ChatGPT-3.5, Google’s Bard, and Anthropic’s Claude, each using their September 2023 version, and found that detection accuracy varied by model and task, with ChatGPT achieving up to 83% accuracy in some cases [16]. However, these findings have not been peer-reviewed and do not include the more recent GPT-4 architecture, which may produce outputs that are even more difficult to distinguish from human-written text.

In this preliminary study, we evaluated whether ChatGPT-4.0 can determine if a scientific abstract was written by itself or by human authors. We assessed both classification accuracy and reproducibility over two rounds of evaluation. Given the increasing fluency and human-likeness of LLMs, we hypothesized that ChatGPT-4.0 would not be able to reliably or consistently distinguish its own output from human-written abstracts. Determining whether LLMs like ChatGPT can reliably identify AI-generated content is increasingly relevant, as such capabilities could contribute to transparency and accountability in academic publishing.

## Materials and methods

We selected 10 general internal medicine journals with the highest Journal Citation Reports (JCR) 2023 impact factors, meeting the following criteria: (i) publication of original research and/or systematic reviews; (ii) use of structured abstracts for both; (iii) existence since at least January 2000. From each journal, we randomly selected 10 articles (either original research or systematic reviews) published between January and December 2000, a period predating modern AI tools. We did not stratify by article type. The list of journals is provided in the Appendix.

We then used ChatGPT-4.0 to generate a structured abstract for each article with the following prompt: "Please write a structured abstract of up to 300 words for the article provided as a PDF file, using the following sections: Background, Methods, Results, and Conclusion". Before submitting the article to ChatGPT-4.0, we removed the original abstract from the PDF to ensure that the generated abstract was based solely on the body of the article. ChatGPT-4.0 was chosen for its ability to read PDF files, ensuring access to all tables, figures, and textual content. In parallel, we collected the original abstracts written by the human authors.

This resulted in 100 AI-generated abstracts and 100 human-written abstracts. Each of the 200 abstracts was then submitted to ChatGPT-4.0 for authorship classification, using the prompt: "How likely is it that this abstract was written by the authors or by ChatGPT? Rate from 0 (definitely authors) to 10 (definitely ChatGPT), with 5 meaning unsure". We used a 0-10 scale to allow for nuanced confidence ratings, offering greater granularity than commonly used Likert scales (e.g., five- or seven-point scales), with 0 representing "definitely human", 10 "definitely ChatGPT", and 5 serving as a natural midpoint explicitly defined as "unsure". All prompts were submitted via the ChatGPT Plus interface (April 2025 version), which uses a default temperature of 1.0. No system message was used; only the user prompt was provided.

Each abstract was assessed twice to evaluate intra-model consistency, leading to 400 total evaluations (200 abstracts × 2 rounds). Both the generation of abstracts and the authorship classification tasks were performed in April 2025. We chose to focus on abstracts in this preliminary study because they are standardized, concise, and widely used in scientific communication, making them a practical starting point for evaluating ChatGPT’s ability to detect AI-generated text.

Statistical analysis

All ratings were recorded on a 0-10 scale, where scores from 0 to 4 were classified as indicating human authorship, scores from 6 to 10 were classified as AI-generated, and a score of 5 was considered uncertain and thus categorized as non-classified. Each prediction was then categorized as either correct (c) if the predicted authorship matched the true authorship, incorrect (i) if the prediction was incorrect, or unclassified (u) if the score was 5 and no classification was made.

To evaluate performance, we calculated three metrics commonly used in prior studies [17-21]. The total error rate (ErrorCoded) was defined as the proportion of incorrect and non-classified predictions relative to all predictions: (i + u) / (c + i + u). The misclassification rate excluding unclassified cases (ErrorCodedWithoutNA) was calculated as i / (c + i). Finally, the proportion of non-classified cases (NACoded) was computed as u / (c + i + u). We used the chi-squared test to compare the distribution of classification outcomes across actual authorship categories in each round.

Agreement between the two rounds of classification was assessed using Cohen’s kappa, both unweighted and weighted (with linear weights) to account for the ordinal nature of the 0-10 scale. Kappa values were interpreted according to the scale proposed by Fleiss, where values below 0.40 indicate poor agreement, 0.40-0.75 reflect fair to good agreement, and values above 0.75 indicate excellent agreement [22]. We also examined the distribution of classification scores across actual authorship categories (human vs. AI-generated abstracts) and tested for statistically significant differences using the Wilcoxon rank-sum test.

All statistical analyses were performed using Stata version 15.1 (StataCorp, College Station, TX).

## Results

Classification outcomes for both rounds are shown in Table 1.

**Table 1:** Confusion matrix and performance metrics for ChatGPT-4.0 (100 abstracts written by humans and 100 abstracts generated by ChatGPT). ^1 ^Chi-squared test comparing the classification distributions between AI- and human-written abstracts. Abstracts were classified as "human" if the score was between 0 and 4, "AI" if the score was between 6 and 10, and "unclassified" if the score was 5. Performance metrics: errorCoded (i.e., proportion of misclassifications and non-classifications) and errorCodedWithoutNA (i.e., proportion of misclassifications) = 0.490 in round 1 and 0.475 in round 2; naCoded (i.e., proportion of non-classifications) = 0 in both rounds. Inter-round agreement: 56 abstracts were classified as AI in both rounds, 77 as human in both rounds, 40 were AI in round 1 and human in round 2, and 27 showed the reverse pattern. Overall agreement = 66.5% (95% CI: 59.9%–73.1%, p-value < 0.001). Cohen’s Kappa = 0.33 (95% CI: 0.19–0.46, p-value < 0.001).

	Abstract classified as AI, n (%)	Abstract classified as human, n (%)	Abstract unclassified, n (%)	p-value^1^
ChatGPT-4.0 (Round 1)				0.78
Abstract authored by ChatGPT (n = 100)	49 (49%)	51 (51%)	0	
Abstract authored by humans (n = 100)	47 (47%)	53 (53%)	0	
ChatGPT-4.0 (Round 2)				0.47
Abstract authored by ChatGPT (n = 100)	44 (44%)	56 (56%)	0	
Abstract authored by humans (n = 100)	39 (39%)	61 (61%)	0	

ChatGPT-4.0 misclassified nearly half of the abstracts in both rounds, with no statistically significant difference between the distributions of classification outcomes across actual authorship categories (p-value = 0.78 in round 1 and 0.47 in round 2). No abstract received a score of 5, meaning that all were classified as either human-written or AI-generated. Consequently, the non-classification rate was zero.

Agreement between the two rounds of classification was poor. Although 67% of abstracts received consistent classifications (95% CI: 60%-73%; p-value < 0.001), Cohen’s kappa was only 0.33 (95% CI: 0.19-0.46; p-value < 0.001), indicating low inter-round reliability.

**Figure 1:**
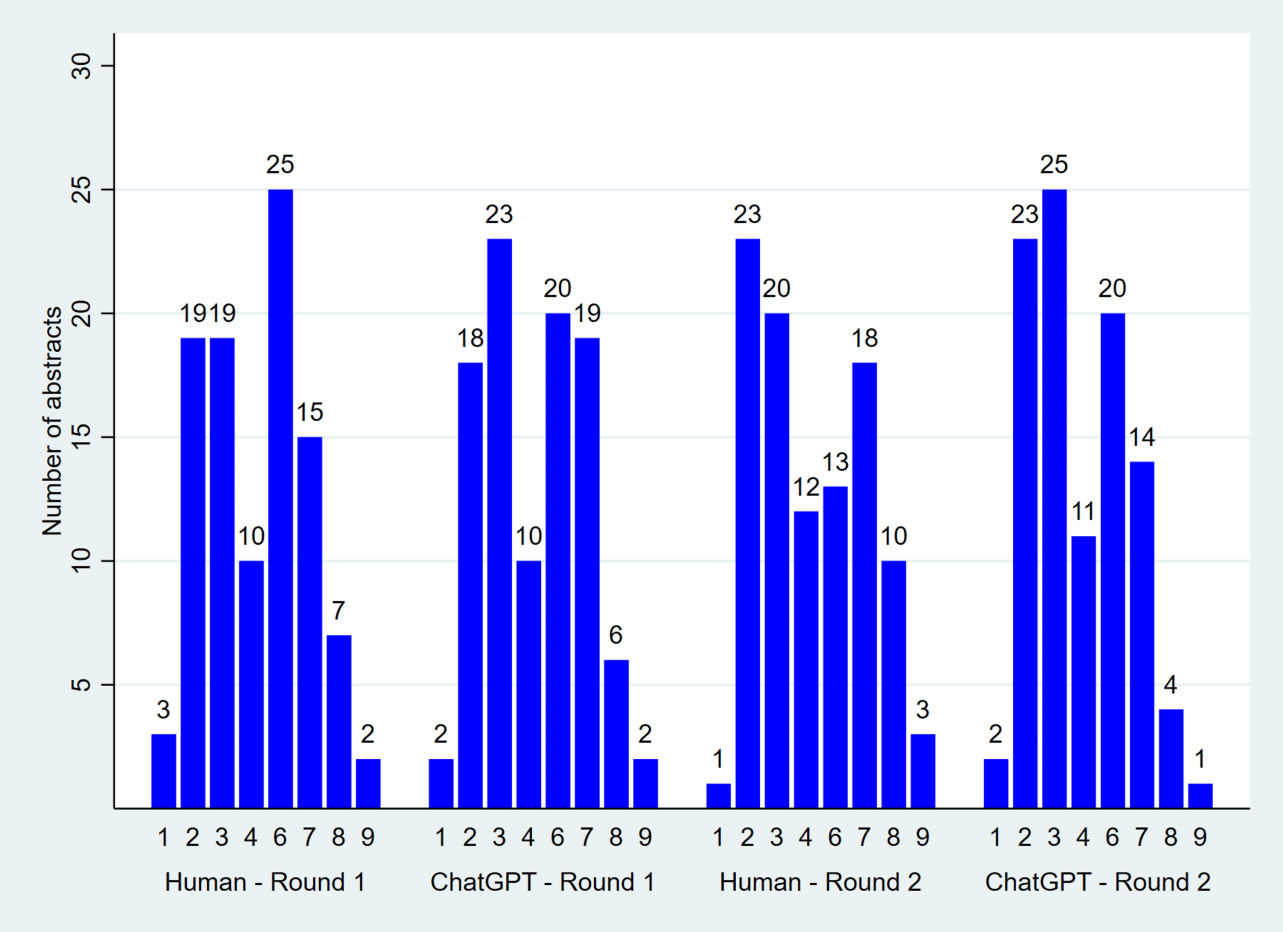
Distribution of classification scores (0–10) assigned by ChatGPT-4.0, by actual author (human vs. ChatGPT), and round of evaluation. Scores range from 0 (definitely human-written) to 10 (definitely AI-generated); each abstract was evaluated twice. Median (IQR) scores were 4 (3–6) vs. 4 (3–7) in round 1 (p-value = 0.93), and 4 (3–7) vs. 3.5 (2.5–6) in round 2 (p-value = 0.21), for AI-generated and human-written abstracts, respectively (Wilcoxon rank-sum test). Inter-round agreement: exact agreement = 23.5% (95% CI: 17.6%–29.4%, p-value < 0.001), weighted Cohen’s Kappa = 0.24 (95% CI: 0.15–0.34, p-value < 0.001).

Score distributions (from 0 to 10) overlapped considerably between AI-generated and human-written abstracts in both rounds (Figure 1). No significant difference was found between the groups in either round. Median (IQR) scores were 4 (3-6) vs. 4 (3-7) in round 1 (p-value = 0.93), and 4 (3-7) vs. 3.5 (2.5-6) in round 2 (p-value = 0.21), for AI-generated and human-written abstracts, respectively.

Exact numerical agreement between rounds occurred in less than one-quarter of cases: 24% (95% CI: 18%-29%, p-value < 0.001). Weighted Cohen’s kappa for the full 0-10 scale indicated low reproducibility in scoring: 0.24 (95% CI: 0.15-0.34, p-value < 0.001).

## Discussion

Summary of key findings

This study evaluated whether ChatGPT-4.0 could determine whether a scientific abstract had been written by itself or by a human author. Across two rounds of evaluation, the model misclassified nearly half of the 200 abstracts, with no significant differences in score distributions between AI- and human-written texts. Agreement between rounds was low: Cohen’s kappa for binary classification was 0.33, and the weighted kappa on the 0-10 scale was 0.24, both indicating poor reliability. These findings suggest that ChatGPT-4.0 lacks the ability to accurately and consistently identify the origin of scientific text.

Comparison with the literature

These findings align with a growing body of literature suggesting that AI-generated scientific writing is increasingly difficult to distinguish from human-authored content [4,10-16]. There are four main approaches described in the literature to assess whether a text was written by AI: human reviewer judgment, external detection tools (which may incorporate linguistic analysis), standalone linguistic feature analysis, and self-detection by the model itself. We present one representative study for each approach in this section, each illustrating the difficulty of reliably identifying AI-generated scientific text.

Gao et al. (2023) conducted a blinded study in which human reviewers evaluated scientific abstracts written by ChatGPT-3 [4]. They found that reviewers correctly identified AI-generated abstracts only slightly better than chance. Similarly, Weber-Wulff et al. (2023) tested 14 automated tools designed to detect AI-generated text and found that their performance was limited [10]. None of the tools achieved over 80% accuracy, and only five exceeded 70%. Other studies have explored linguistic features as potential markers of AI authorship [13,23-27]. For example, Gehrmann et al. (2019) introduced the GLTR tool, which analyzes the statistical likelihood of word choices to highlight differences in text predictability between AI- and human-written content [23]. AI-generated texts tend to use more high-probability (predictable) words, while human texts are more varied. In their study, GLTR helped untrained users better detect AI-generated content. However, it is not a standalone detector, relies on manual interpretation, and was developed using GPT-2, limiting its applicability to more recent models like GPT-4.

In contrast to these external tools, our study assessed whether ChatGPT-4.0 could self-detect authorship, an approach that assumes some form of internal trace or stylistic recognition. The results suggest that such an internal signal does not exist, at least in the context of structured scientific abstracts. Although ChatGPT had access to full-text PDFs for abstract generation, it did not demonstrate any measurable advantage in classifying its own output. Furthermore, the model’s classification scores were unstable across rounds, suggesting that its predictions were driven more by randomness or linguistic surface features than by any consistent authorship cues.

To our knowledge, only one other study has used a similar self-detection approach. In a preprint that has not been peer-reviewed, Caiado and Hahsler (2023) introduced the concept of "AI content self-detection", evaluating whether generative models like ChatGPT-3.5, Bard, and Claude could recognize texts they had written [16]. In their experiments, models were asked to classify whether short texts had been generated by themselves, without receiving any prior examples or fine-tuning for this specific task. ChatGPT achieved up to 83% accuracy, substantially higher than in our study. However, the tasks differ in important ways. Their dataset consisted of brief, general-topic essays generated from simple and uniform prompts, which likely made the outputs more predictable and easier for the model to recognize. Furthermore, their analysis was limited to earlier versions of language models and did not include GPT-4, whose outputs may be more difficult to distinguish from human writing. These differences likely account for the gap in performance and highlight the need to test self-detection methods in more complex, domain-specific, and realistic contexts such as scientific publishing.

Implications for practice and research

These findings have several practical implications. First, they highlight the limitations of using LLMs like ChatGPT-4.0 as tools for identifying AI-generated content in scientific writing. As journals adopt or consider AI-detection protocols, our results caution against using ChatGPT itself for this purpose. Second, these findings underscore the challenge facing researchers, peer reviewers, and editors, who may encounter AI-generated text that is indistinguishable from human writing. Third, this study supports the call for transparent disclosure policies and technical tools to track the use of generative AI in research workflows. Future efforts may be more effective by combining AI-detection tools with contextual metadata, such as submission timestamps, version history, or editing logs, which can offer additional clues about how a document was produced. Requiring authors to explicitly disclose any use of generative AI would further strengthen transparency and accountability in research workflows.

Strengths and limitations

This study has several notable strengths. It is, to our knowledge, the first peer-reviewed study to evaluate ChatGPT-4.0’s ability to detect its own outputs in the context of scientific abstracts. It uses real-world, domain-specific material drawn from high-impact journals and applies a standardized scoring scale with explicit prompts. By testing self-detection rather than relying on human judges or third-party tools, the study also introduces a novel evaluation framework relevant for research on AI transparency and epistemic trust. These elements contribute to its originality and potential impact in the fields of medical publishing and AI ethics.

However, several limitations should be acknowledged. First, we included only abstracts from internal medicine journals published in 2000, which may limit the generalizability of our findings to other disciplines, time periods, or types of scientific texts. Results might differ if full-text articles were used, as they provide more context, complexity, and stylistic variation. Second, although we analyzed the full 0-10 scoring scale to capture more nuanced differences between ChatGPT-written and original abstracts, the scale itself, including the dichotomous thresholds we applied (0-4 = human, 6-10 = AI), was not externally validated. This limits the interpretability of the numerical scores and classification outcomes. Interestingly, although the prompt allowed for uncertainty (score = 5), ChatGPT never used this option in either round. This suggests a tendency to avoid midpoint responses, possibly due to an inherent model tendency toward forced classification. While the score distributions overlapped substantially between human- and AI-written abstracts, we interpret this not as a flaw in the scoring method, but as evidence that ChatGPT-4.0 is unable to detect meaningful differences between the two groups. Future studies could explore alternative rating instructions, comparisons with human raters or external detection tools, and other response formats to assess whether more consistent scoring patterns can be obtained. Third, we relied on a single model version (GPT-4.0) and a single prompt. While the exact prompt is reported in the Methods section, it was not compared to alternative formulations, and different phrasings might have influenced the model’s classification behavior. Finally, using ChatGPT as both the generator and the classifier raises potential concerns about circularity and shared model biases.

## Conclusions

ChatGPT-4.0 is not reliable in identifying whether a scientific abstract was written by itself or by a human author. Its accuracy was low, and its classifications were inconsistent across repeated evaluations. Given the increasing use of AI tools in scientific writing, these findings underscore the need for more robust and transparent methods to detect AI-generated content in academic publishing. Future research should investigate whether similar results are observed when using full-text articles, which may contain richer contextual and stylistic cues. Until such studies are conducted, we cannot assume that AI models, even those that generate the content, are capable of reliably identifying AI authorship. Addressing this limitation will be essential for developing trustworthy detection strategies and maintaining the integrity of scientific communication.

## Appendices

**Table 2:** Journals included in the study, with 2023 Journal Citation Reports (JCR) impact factor, and number of articles selected per journal. ¹ The Archives of Internal Medicine is now known as JAMA Internal Medicine, and the impact factor corresponds to the current title.

Journal name	2023 Impact factor	Number of articles selected for the study
The Lancet	98.4	10
New England Journal of Medicine	96.3	10
The BMJ	93.7	10
JAMA	63.5	10
Archives of Internal Medicine¹	22.3	10
Annals of Internal Medicine	19.6	10
CMAJ (Canadian Medical Association Journal)	12.9	10
Journal of Travel Medicine	9.1	10
Journal of Internal Medicine	9.0	10
Mayo Clinic Proceedings	6.9	10

## References

[1] Kung TH, Cheatham M, Medenilla A (2023). Performance of ChatGPT on USMLE: potential for AI-assisted medical education using large language models. PLOS Digit Health.

[2] Sallam M (2023). ChatGPT utility in healthcare education, research, and practice: systematic review on the promising perspectives and valid concerns. Healthcare (Basel).

[3] Shen Y, Heacock L, Elias J, Hentel KD, Reig B, Shih G, Moy L (2023). ChatGPT and other large language models are double-edged swords. Radiology.

[4] Gao CA, Howard FM, Markov NS, Dyer EC, Ramesh S, Luo Y, Pearson AT (2023). Comparing scientific abstracts generated by ChatGPT to real abstracts with detectors and blinded human reviewers. NPJ Digit Med.

[5] Rao A, Pang M, Kim J (2023). Assessing the utility of ChatGPT throughout the entire clinical workflow: development and usability study. J Med Internet Res.

[6] Dave T, Athaluri SA, Singh S (2023). ChatGPT in medicine: an overview of its applications, advantages, limitations, future prospects, and ethical considerations. Front Artif Intell.

[7] Else H (2023). Abstracts written by ChatGPT fool scientists. Nature.

[8] Thorp HH (2023). ChatGPT is fun, but not an author. Science.

[9] Stokel-Walker C (2023). ChatGPT listed as author on research papers: many scientists disapprove. Nature.

[10] Weber-Wulff D, Anohina-Naumeca A, Bjelobaba S (2023). Testing of detection tools for AI-generated text. Int J Educ Integr.

[11] Walters WH (2023). The effectiveness of software designed to detect AI-generated writing: a comparison of 16 AI text detectors. Open Inf Sci.

[12] Clark E, August T, Serrano S, Haduong N, Gururangan S, Smith NA (2021). All that’s ‘human’ is not gold: evaluating human evaluation of generated text. Proceedings of the 59th Annual Meeting of the Association for Computational Linguistics and the 11th International Joint Conference on Natural Language Processing (Volume 1: Long Papers).

[13] Else H (2021). 'Tortured phrases' give away fabricated research papers. Nature.

[14] Al-Rawas M, Qader OA, Othman NH (2025). Identification of dental related ChatGPT generated abstracts by senior and young academicians versus artificial intelligence detectors and a similarity detector. Sci Rep.

[15] Nabata KJ, AlShehri Y, Mashat A, Wiseman SM (2025). Evaluating human ability to distinguish between ChatGPT-generated and original scientific abstracts. Updates Surg.

[16] Caiado AJA, Hahsler M (2023). AI content self-detection for transformer-based large language models. [PREPRINT]. arXiv.

[17] Sebo P (2024). What is the performance of ChatGPT in determining the gender of individuals based on their first and last names?. JMIR AI.

[18] Sebo P (2021). Performance of gender detection tools: a comparative study of name-to-gender inference services. J Med Libr Assoc.

[19] Santamaría L, Mihaljević H (2018). Comparison and benchmark of name-to-gender inference services. PeerJ Comput Sci.

[20] Sebo P (2023). How well does NamSor perform in predicting the country of origin and ethnicity of individuals based on their first and last names?. PLoS One.

[21] Wais K (2016). Gender prediction methods based on first names with GenderizeR. R J.

[22] Fleiss JL, Levin B, Paik MC (1981). Statistical Methods for Rates and Proportions. Second Edition. https://onlinelibrary.wiley.com/doi/book/10.1002/0471445428.

[23] Gehrmann S, Strobelt H, Rush A (2019). GLTR: Statistical detection and visualization of generated text. Proceedings of the 57th Annual Meeting of the Association for Computational Linguistics: System Demonstrations.

[24] Akinwande M, Adeliyi O, Yussuph T (2024). Decoding AI and human authorship: nuances revealed through NLP and statistical analysis. Int J Cybern Inform.

[25] Amirjalili F, Neysani M, Nikbakht A (2024). Exploring the boundaries of authorship: a comparative analysis of AI-generated text and human academic writing in English literature. Front Educ.

[26] Goulart L, Matte ML, Mendoza A, Alvarado L, Veloso I (2024). AI or student writing? Analyzing the situational and linguistic characteristics of undergraduate student writing and AI-generated assignments. J Second Lang Writ.

[27] Rujeedawa MIH, Pudaruth S, Malele V (2025). Unmasking AI-generated texts using linguistic and stylistic features. Int J Adv Comput Sci Appl.

